# Impaired self-awareness after traumatic brain injury: inter-rater reliability and factor structure of the Dysexecutive Questionnaire (DEX) in patients, significant others and clinicians

**DOI:** 10.3389/fnbeh.2014.00352

**Published:** 2014-10-10

**Authors:** Brian E. McGuire, Todd G. Morrison, Lynne A. Barker, Nicholas Morton, Judith McBrinn, Sheena Caldwell, Colin F. Wilson, John McCann, Simone Carton, Mark Delargy, Jane Walsh

**Affiliations:** ^1^School of Psychology, National University of Ireland GalwayGalway, Ireland; ^2^Department of Psychology, University of SaskatchewanSaskatoon, SK, Canada; ^3^Department of Psychology, Sheffield Hallam UniversitySheffield, UK; ^4^Neurorehabilitation Services, Doncaster Rotherham and South Humber NHS Foundation TrustDoncaster, UK; ^5^Regional Acquired Brain Injury Unit, Musgrave Park HospitalBelfast, UK; ^6^National Rehabilitation HospitalDublin, Ireland

**Keywords:** brain injury, dysexecutive, reliability, factor analysis, care giver

## Abstract

**Aims**: This study sought to address two questions: (1) what is the inter-rater reliability of the Dysexecutive Questionnaire (DEX) when completed by patients, their significant others, and clinicians; and (2) does the factor structure of the DEX vary for these three groups?

**Methods**: We obtained DEX ratings for 113 patients with an acquired brain injury from two brain injury services in the UK and two services in Ireland. We gathered data from two groups of raters—“significant others” (DEX-SO) such as partners and close family members and “clinicians” (DEX-C), who were psychologists or rehabilitation physicians working closely with the patient and who were able to provide an opinion about the patient’s level of everyday executive functioning. Intra-class correlation coefficients and their 95% confidence intervals were calculated between each of the three groups (self, significant other, clinician). Principal axis factor (PAF) analyses were also conducted for each of the three groups.

**Results**: The factor analysis revealed a consistent one-factor model for each of the three groups of raters. However, the inter-rater reliability analyses showed a low level of agreement between the self-ratings and the ratings of the two groups of independent raters. We also found low agreement between the significant others and the clinicians.

**Conclusion**: Although there was a consistent finding of a single factor solution for each of the three groups, the low level of agreement between significant others and clinicians raises a question about the reliability of the DEX.

## Introduction

Impaired self-awareness is a common cognitive deficit after traumatic brain injury and can lead to problems with self-monitoring and behavioral self-regulation (McBrinn et al., 2008). These problems may contribute to difficulties undertaking many everyday functions such as engaging in interpersonal communication, budgeting, household chores and carrying out vocational activities (Godbout et al., 2005). The cognitive capacities associated with self-awareness and self-regulation are considered to be part of the executive system in the frontal lobes of the brain. Executive functions include cognitive operations that contribute to the ability to initiate, inhibit and integrate other functions, simultaneously termed supervisory, attentional or control processes (Shallice and Burgess, 1991; Miyake et al., 2000; Stuss and Alexander, 2007).

A cluster of symptoms associated with these functions is thought to present in “dysexecutive syndrome”, the central component of the cluster being impairment in self-awareness and self-regulation (Morton and Barker, 2010). This impairment is assumed to arise from damage to critical areas of the brain that are integral to behavioral self-regulation, typically the frontal lobes. There is, however, growing recognition that self-awareness is a highly complex and multifaceted process that is not exclusive to the frontal lobes. Efforts to identify specific brain areas that may be responsible for self-monitoring and self-regulation have led researchers to acknowledge that multiple pathways may be involved—impaired self-awareness does not appear to be linked exclusively to focal or generalized brain damage or to specific neurocognitive test profiles (Philippi et al., 2012; Caldwell et al., 2014; Ham et al., 2014).

While the study of the underlying processes involved in impaired self-regulation continues, clinicians agree that the capacity for self-monitoring and behavioral self-regulation is important to successful rehabilitation outcomes after brain injury (Winkens et al., 2014). To that end, clinicians are reliant on existing psychometric tools for identifying and quantifying impaired self-regulation. However, measurement of executive ability is challenging as executive function tests are not process-pure—they will invariably and unavoidably involve other non-executive functions that may be variously spared or compromised after brain injury (Barker et al., 2010). One commonly used measure of the behavioral manifestation of dysexecutive impairment is the Dysexecutive Questionnaire (DEX; Wilson et al., 1997). The DEX is purported to be an ecologically valid test; that is, it provides an estimation of executive function as applied to everyday life challenges. The interpretation of the DEX score is based on the difference between the client’s self-report and the report of another person who knows the client well, with any resultant discrepancy assumed to reflect a lack of self-awareness in the brain-injured person.

As a relatively quick and easy questionnaire to complete, the DEX offers an appealing method of quantifying a complex neuropsychological process. However, the utility of the test relies on two important premises: (1) the third party respondent can give a true and accurate account of the injured person’s functioning; and (2) the psychometric validity of the measurement tool is constant across users (i.e., both client and independent rater “versions” are measuring the same construct or factor[s]). Each of these premises is considered briefly.

Regarding the first premise, there is certainly evidence that patient self-reports differ from the reports of their significant others. This finding is not unexpected as the DEX is designed to identify discrepancies in scores that may reflect impairment in self-awareness in people following brain injury. However, some evidence suggests that independent raters may not respond in a similar way about the same person. For example, a study of the inter-rater reliability of the ratings of family members found a low level of agreement among three independent raters reporting about the same individual with a brain injury (Barker et al., 2011). The authors concluded that all raters do not respond in a comparable manner and, thus, it would be erroneous for clinicians to assume that DEX ratings by significant others are always accurate (Barker et al., 2011). The problem of ascertaining whether a rating by a family member is accurate is more complex than it may appear. For example, if one does not use independent ratings of the level of impaired awareness of the person with brain injury, then the other main source of information is objective neuropsychological data. However, the situation here is far from clear—there is not a direct correlation between overall severity of cognitive impairment and level of impaired self-awareness or between scores on specific tests of executive function and level of impaired self-awareness (Barker et al., 2004). Thus, there are ongoing questions regarding the precise nature of impaired self-awareness, its link to overall executive functioning, and the extent to which the construct can be measured by existing questionnaire-based tools.

With respect to the second premise of whether the DEX self-rated questionnaire measures the same construct(s) as the DEX completed by independent others, several studies have examined the factor structure of the DEX focusing on DEX self-ratings. Variable findings have been obtained. For instance, a study using a large community sample of more than 1100 people identified a single underlying factor (Gerstorf et al., 2008). Conversely, a study using non-clinical (*N* = 293) and clinical (*N* = 49) samples found a 4-factor solution with factors best described as inhibition, intention, social regulation, and abstract problem-solving (Mooney et al., 2006). A 4-factor model also was identified by Bodenburg and Dopslaff (2008); however, based on different loadings, their interpretations of these factors were: initiating and sustaining actions, impulse control, psychophysical and mental excitability, and social conventions. A study of the factor structure of the DEX in the context of normal aging (Amieva et al., 2003) identified a 5-factor solution: intentionality, interference management, inhibition, planning, and social regulation. Thus, substantial variability is evident in the dimensionality of the DEX.

Only one previous study has tested the factor structure of the DEX amongst independent raters. Using the significant others of 46 adults with varying neurological conditions, that study obtained a 3-factor solution described as behavioral inhibition, goal-directed behavior/intentionality, and executive memory/cognition (Chaytor and Schmitter-Edgecombe, 2007). No studies have yet examined the factor structure of the DEX when completed by independent raters who are reporting about the degree of impairment associated with acquired brain injury. Further, no previous study has compared the factor structure of the DEX when completed by two or more independent raters in relation to the same patient. The fundamental questions addressed by this study are: (1) what are the levels of inter-rater consistency when the DEX is completed by patients, significant others, and clinicians; and (2) does the dimensionality of the DEX vary as a function of the individuals completing it (e.g., client vs. clinician)?

## Method

### Measures

The Behavioral Assessment of the Dysexecutive Syndrome (BADS) is considered an ecologically valid, multidimensional measure of executive function comprising six sub-tests and a questionnaire which probes symptoms of Dysexecutive syndrome, called the DEX (Wilson et al., 1997). The DEX is a 20-item questionnaire which the authors describe as having three factors assessing everyday changes in cognition, emotion and behavior after an acquired brain injury or other brain trauma. The DEX is completed by the patient (self-rating: DEX-S) and by a person who knows the patient well (independent rater). In this study, we gathered data from two groups of independent raters—“significant others” (DEX-SO) such as partners and close family members and “clinicians” (DEX-C), who were psychologists or rehabilitation physicians working closely with the patient and who were able to provide an opinion about the patient’s level of everyday executive functioning.

Ethical Approval: Each participant and their significant other provided consent to take part and each of the participating services received ethical approval from their local institutional research ethics committee.

### Participants

The number of patients included in this study was 113 (87 males, *M* age = 37.77, *SD* = 12.76; 26 females, *M* age = 38.96, *SD* = 12.06) from two brain injury services in the UK and two services in Ireland. The participants were identified by the service managers and clinicians by virtue of being a client of the service and meeting the inclusion criteria. Inclusion criteria for the study were: 18 years or older, had experienced an acquired brain injury, had sufficient cognitive and physical ability to give informed consent to participate, able to read and respond to the questionnaires. Exclusion criteria were major psychiatric illness or cognitive impairment of such severity that would prevent the ability to consent and/or to respond to the questionnaires. None of those identified by the services as potentially suitable participants refused to participate. In each center, an unspecified number of patients were deemed by the clinician or service manager to not meet the inclusion criteria. The sample in the study would, in the authors’ view, be considered typical of those accessing brain injury support services in the UK and Ireland with moderately severe brain injury. All were in the post-acute phase of rehabilitation, typically receiving support services focused on optimizing independent functioning. The mean duration of injury was 57.49 months (*SD* = 44.24), with minimal and maximal time periods of 10 months and 168 months, respectively. The median for duration was 36 months (25th percentile = 24 months; 75th percentile = 84 months). Type of injury data indicated that an overwhelming majority of clients had experienced traumatic head injuries (95%). Finally, with respect to current occupation, the most commonly selected options were: currently unemployed (24.1%), supported training/employment (20.4%) and retired (20.4%).

Data also were collected from caregiver/significant other (DEX-SO; *N* = 101) and clinician (DEX-C; *N* = 64) raters. Within the caregiver/significant others group, parents (*n* = 40), spouses (*n* = 38), siblings (*n* = 9), adult offspring (*n* = 6), friends (*n* = 5), and other family members (*n* = 2) were represented.

## Results

Table 1 provides descriptive statistics and reliability coefficients for the DEX-S (i.e., self-ratings) as well as the DEX-SO and the DEX-C.

**Table 1:** **Means, standard deviations, and Cronbach’s alpha coefficients for DEX scales**.

Questionnaire category	*N*	Mean
		Standard deviation
		Cronbach’s alpha coefficient (95% confidence intervals)
DEX-Self	113	29.08
		16.54
		0.93 (0.90–0.94)
DEX-Significant other	101	33.63
		19.28
		0.95 (0.94–0.96)
DEX-Clinician	64	23.81
		18.78
		0.97 (0.96–0.98)

Cronbach’s alpha coefficients and their 95% confidence intervals suggest excellent scale score reliability within each respondent group. However, upper bound estimates, particularly for the DEX-SO and DEX-C, suggest that item redundancy may be of concern (Streiner, 2003). As noted in previous research, DEX-SO scores were higher than DEX-S ratings, although this difference was not statistically significant (LSD, *p* = 0.07). DEX-C scores were lowest of all and differed significantly from DEX-SO scores (LSD, *p* = 0.001).

Intra-class correlation coefficients and their 95% confidence intervals were calculated between DEX-S and DEX-SO items; DEX-S and DEX-C items; and DEX-SO and DEX-C items. This analysis permits one to determine the degree of consistency between self-, significant other, and clinician ratings, with ICC values >0.74 representing an excellent level of agreement; values between 0.60 and 0.74 reflecting good agreement; and values between 0.40 and 0.59 representing fair agreement. Absolute agreement ICCs were estimated using a one-way random effects model (see Table 2).

**Table 2:** **Intra-class correlation coefficients and their 95% confidence intervals between DEX-Self (DEX-S), DEX-Significant Other (DEX-SO) and DEX-Clinician (DEX-C)**.

DEX item	*DEX-S and DEX-SO*	*DEX-S and DEX-C*	*DEX-SO and DEX-C*
1. Has problems understanding what other people mean unless they keep things simple and straightforward	0.35 (0.16–0.51)	0.00 (−0.25–0.24)	0.19 (−0.08–0.44)
2. Acts without thinking, doing the first thing that comes to mind	0.45 (0.28–0.59)	0.20 (−0.05–0.42)	0.24 (−0.03–0.48)
3. Sometimes talks about events or details that never actually happened, but s/he believes did happen	0.30 (0.11–0.47)	0.09 (−0.15–0.33)	0.51 (0.28–0.69)
4. Has difficulty thinking ahead or planning for the future	0.43 (0.26–0.58)	0.07 (−0.18–0.31)	0.49 (0.26–0.67)
5. Sometimes gets over-excited about things and can be a bit “over-the-top” at these times	0.40 (0.23–0.55)	0.13 (−0.12–0.36)	0.48 (0.24–0.66)
6. Gets events mixed up with each other, and gets confused about the correct order of events	0.34 (0.16–0.50)	0.19 (−0.05–0.42)	0.28 (0.01–0.51)
7. Has difficulty realizing the extent of his/her problems and is unrealistic about the future	0.20 (0.01–0.38)	0.00 (−0.25–0.24)	0.31 (0.05–0.54)
8. Seems lethargic, or unenthusiastic about things	0.33 (0.14–0.49)	0.13 (−0.11–0.37)	0.04 (−0.23–0.31)
9. Does or says embarrassing things when in the company of others	0.49 (0.33–0.62)	0.26 (0.02–0.47)	0.34 (0.08–0.56)
10. Really wants to do something 1 min, but couldn’t care less about it the next	0.56 (0.41–0.68)	0.29 (0.05–0.50)	0.35 (0.09–0.56)
11. Has difficulty showing emotion	0.35 (0.16–0.51)	0.16 (−0.09–0.39)	0.11 (−0.16–0.37)
12. Loses his/her temper at the slightest thing	0.54 (0.39–0.67)	0.11 (−0.14–0.35)	0.28 (0.01–0.51)
13. Seems unconcerned about how s/he should behave in certain situations	0.46 (0.29–0.60)	0.19 (−0.06–0.41)	0.54 (0.31–0.71)
14. Finds it hard to stop repeating saying or doing things once started	0.50 (0.34–0.63)	0.01 (−0.24–0.25)	0.40 (0.15–0.61)
15. Tends to be very restless and “can’t sit still” for any length of time	0.40 (0.23–0.55)	0.06 (−0.18–0.30)	0.24 (−0.03–0.48)
16. Finds it difficult to stop doing something even if s/he knows s/he shouldn’t	0.43 (0.26–0.58)	0.25 (0.00–0.46)	0.23 (−0.04–0.47)
17. Will say one thing, but do something different	0.46 (0.29–0.60)	0.15 (−0.10–0.38)	0.34 (0.07–0.56)
18. Finds it difficult to keep his/her mind on something, and is easily distracted	0.29 (0.10–0.46)	0.18 (−0.06–0.41)	0.32 (0.06–0.55)
19. Has trouble making decisions, or deciding what s/he wants to do	0.50 (0.33–0.63)	0.26 (0.02–0.47)	0.23 (−0.04–0.47)
20. Is unaware of, or unconcerned about, how others feel about his/her behavior	0.38 (0.20–0.54)	0.29 (−0.05–0.50)	0.27 (0.01–0.51)

The average level of agreement between self- and significant other ratings was 0.41 (*SD* = 0.09). The averages for self- and clinician ratings and significant other and clinician ratings were 0.15 (*SD* = 0.09) and 0.31 (*SD* = 0.13), respectively. *Post-hoc* testing revealed that these averages differed significantly: self and significant other vs. self and clinician (LSD, *p* < 0.001); self and significant other vs. significant other and clinician (LSD, *p* < 0.01); self and clinician vs. significant other and clinician (LSD, *p* < 0.001). Importantly, the average level of agreement between self and significant other ratings was at the bottom end of the stratum denoting “fair agreement”. The remaining averages were poor. These findings suggest there is only nominal consistency in ratings on the DEX among patients, their caregivers, and clinicians.

To assess the dimensionality of the DEX when completed by patients, significant others and clinicians, three principal axis factor (PAF) analyses were conducted. This factor analytic technique is recommended when data have the potential to be non-normally distributed (Finch and West, 1997). Diagnostics, such as the Kaiser-Meyer-Olkin (KMO) measure of sampling adequacy and Bartlett’s test of sphericity, were conducted for each PAF analysis and deemed to be satisfactory (i.e., KMO exceeded 0.90 and Bartlett’s test was statistically significant permitting one to reject the null hypothesis that associations among the DEX items may be represented as an identity matrix). Parallel analysis and inspection of the unrotated factor solution were used to assist with factor retention.

When completed by patients, a one-factor solution appeared to best represent the data (i.e., the unrotated solution revealed that no items loaded uniquely on any factor besides the first one and there was a negligible difference between the eigenvalue associated with factor 2 [1.09] for the random data and the eigenvalue associated with factor 2 [1.49] for the real data). The eigenvalue associated with the first factor was 8.46 (42.29% of the variance accounted for). Factor loadings ranged from 0.33 to 0.78 (see Table 3).

**Table 3:** **Principal axis factor loadings for DEX when completed by patients, significant others, and clinicians**.

DEX item	Patients	Significant Others	Clinicians
1. Has problems understanding what other people mean unless they keep things simple and straightforward	0.406	0.610	0.705
2. Acts without thinking, doing the first thing that comes to mind	0.777	0.647	0.920
3. Sometimes talks about events or details that never actually happened, but s/he believes did happen	0.326	0.579	0.691
4. Has difficulty thinking ahead or planning for the future	0.548	0.720	0.819
5. Sometimes gets over-excited about things and can be a bit “over-the-top” at these times	0.547	0.651	0.813
6. Gets events mixed up with each other, and gets confused about the correct order of events	0.620	0.689	0.833
7. Has difficulty realizing the extent of his/her problems and is unrealistic about the future	0.567	0.773	0.789
8. Seems lethargic, or unenthusiastic about things	0.542	0.630	0.636
9. Does or says embarrassing things when in the company of others	0.644	0.790	0.826
10. Really wants to do something 1 min, but couldn’t care less about it the next	0.769	0.685	0.793
11. Has difficulty showing emotion	0.559	0.587	0.486
12. Loses his/her temper at the slightest thing	0.631	0.722	0.692
13. Seems unconcerned about how s/he should behave in certain situations	0.726	0.831	0.899
14. Finds it hard to stop repeating saying or doing things once started	0.731	0.696	0.788
15. Tends to be very restless and “can’t sit still” for any length of time	0.646	0.651	0.739
16. Finds it difficult to stop doing something even if s/he knows s/he shouldn’t	0.645	0.712	0.860
17. Will say one thing, but do something different	0.769	0.823	0.835
18. Finds it difficult to keep his/her mind on something, and is easily distracted	0.683	0.774	0.696
19. Has trouble making decisions, or deciding what s/he wants to do	0.676	0.712	0.734
20. Is unaware of, or unconcerned about, how others feel about his/her behavior	0.544	0.772	0.924

A similar solution emerged when significant others completed the DEX. Specifically, one factor appeared to best represent the data (eigenvalue = 10.46, accounting for 52.32% of the variance). The factor loadings ranged from 0.58 to 0.83 (see Table 3). Finally, a one-factor solution also was optimal for the clinicians (eigenvalue = 12.54, accounting for 62.72% of the variance). For this group, factor loadings ranged from 0.49 to 0.92.

## Discussion

This study sought to address two questions: (1) what is the inter-rater reliability of the DEX when completed by patients, their significant others, and clinicians; and (2) what is the factor structure of the DEX for these three groups?

Results suggest there is only nominal agreement in item ratings on the DEX among patients, their caregivers, and clinicians. The fact that self-rating and ratings by others is different is not surprising—the very purpose of the measure is to detect a lack of self-awareness in people with brain injury, operationalized as a discrepancy between the patient and their significant other. However, it is concerning that there is a large discrepancy in the ratings of other people who know the patient well: significant others and clinicians attributed quite variable scores across the range of items, indicating a low level of agreement between raters. This finding is in keeping with previous research which showed that the DEX ratings of significant others (mainly family members rather than clinicians) were variable (Barker et al., 2011).

The fact that third party raters can differ quite significantly when reporting about the same individual raises an important question about the reliability of the DEX. It could be suggested that clinician respondents might, by virtue of their professional training, be able to provide a more accurate appraisal of the level of executive function impairment. This is difficult to confirm, however, since clinical judgment is inherently subjective. In the authors’ experience, neuropsychological testing may also not be especially helpful in this regard, as performance on tests of executive function does not always correlate strongly with functional ability (Chaytor et al., 2006; Razani et al., 2007). It may be the case that the best and, perhaps, only reliable way to measure executive function impairment in everyday situations is through a combination of behavioral, task-based measures such as the Multiple Errands Test (Shallice and Burgess, 1991) and a consensus-based response to the DEX where a discussion of the individual items between the respondents may lead to a more accurate description of the problems encountered by the person with brain injury. The feasibility of including ecologically-valid behavioral testing has been improved with the development of virtual reality-based technologies. For example, a virtual reality version of the Multiple Errands Test (Raspelli, 2014) has been developed and offers the potential to measure real-life challenges coupled with the convenience of being able to do the assessment within a clinical setting.

With respect to the dimensionality of the DEX, PAF analysis suggested that a single factor offered the best fit for all three groups. This finding indicates that DEX items are best construed as representing a single construct of executive dysfunction. It should be noted that other researchers have identified a similar factor structure. For instance, using two independent samples of community-dwelling persons, Gerstorf et al. (2008) identified a single factor solution as being optimal for the self-rated version. Specifically, these authors report that “independent of specifying an orthogonal or oblique solution, we found that the eigenvalue for one factor was consistently above 7 whereas four or five other factors could have been extracted but their eigenvalues were only marginally larger than 1” (pp. 432–433). We observed a similar outcome across *three* different categories of respondent: self, significant other, and clinician. To our knowledge, only one other study (Chaytor and Schmitter-Edgecombe, 2007) has examined the factor structure of the DEX when completed by third party respondents (*N* = 46). These researchers identified a five-component solution, with the first three components corresponding well with the inhibition, intentionality, and executive memory factors specified in other psychometric studies assessing the self-rated version. The authors conclude that these three components appear to be replicable whereas components 4 and 5 are, perhaps, idiosyncratic (i.e., components unique to the specific sample being tested). However, the validity of their three-component interpretation may be questioned. First, the authors appear to have relied on the “eigenvalue greater than 1 rule”, which many have argued is among the least accurate methods for identifying factor/component retention (i.e., it often results in over-extraction) (Costello and Osborne, 2005). Second, although the authors do not provide the intercorrelations among the components, based on the study conducted by Gerstorf and associates (Barker et al., 2004), it is possible they are of sufficient magnitude so as to suggest redundancy (i.e., for the factors representing inhibition, intentionality, and executive memory, *r*-values obtained by Gerstorf et al. (2008) ranged from 0.93 to 0.99).

As with all studies, the current investigation possesses certain limitations that warrant discussion. First, the number of participants recruited was modest, especially for the clinician subsample. It should be noted, however, that other researchers have published psychometric assessments of the DEX using similar (or smaller) numbers of participants (e.g., *N* = 20 (Amieva et al., 2003); *N* = 46 (Amieva et al., 2003); *N* = 93 (Bachmann et al., 2008)). Further, MacCallum et al. (1999) demonstrate that “rules of thumb” about sample size are less important than the degree to which a factor solution is characterized by factor over-determination (i.e., the number of indicators per factor, with a common ratio being 5:1) and strong communality values (i.e., the proportion of variance in each item accounted for by the extracted factor[s]). In the current study, communality estimates were variable; strong over-determination was present (i.e., *p* [variable]: *r* [factor] ratio was 20:1); and, for the smallest subsample (clinician group), 19 of the 20 variables had large structure coefficients (>0.60) suggesting that one can be reasonably confident in the reproducibility of the obtained factor solutions. Larger samples are clearly needed, however, if one were to conduct subgroup analyses based on variables such as type of injury, gender of patient, or relationship between patient and significant other (e.g., spouse vs. sibling). Another limitation pertains to the small set of variables that were measured. Gerstorf et al. (2008), for example, assessed a host of individual difference variables including neuroticism, depression, subjective health, trait anxiety, positive and negative affect, and cognitive functioning. In the current study, as only the DEX and a small number of sociodemographic items (e.g., age) were used, the convergent validity of this instrument when completed by patients, significant others and clinicians could not be tested. Future studies might consider the use of alternative methodologies, such as those used in clinical judgment studies (e.g., Bachmann et al., 2008), to look at the cues and weightings used by respondents to arrive at their judgments regarding the presence and extent of any difficulties in executive functioning.

In conclusion, our dimensionality evaluation suggests that the DEX is best construed as a single factor measure of dysexecutive syndrome. The inter-rater reliability analysis suggests that there is a low level of agreement in item ratings on the DEX among patients, their caregivers, and clinicians. The fact that evaluations by two raters are not highly correlated in reference to the same patient raises a question about this element of the reliability of the DEX. While it is well recognized that executive function deficits occur frequently after traumatic brain injury and this is often associated with impaired self-awareness, we are as yet limited in our ability to measure and quantify these impairments. The difficulties arising from measuring deficits in executive function also presents challenges in how best to involve patients in aspects of their own rehabilitation such as patient-determined goals and outcomes (Hogan et al., 2013) when self-awareness is compromised.

## Conflict of interest statement

The authors declare that the research was conducted in the absence of any commercial or financial relationships that could be construed as a potential conflict of interest.
